# Supplementary material for: Major Improvements to the Heliconius melpomene Genome Assembly Used to Confirm 10 Chromosome Fusion Events in 6 Million Years of Butterfly Evolution
Source: G3 (Bethesda). 2016 Jan 15;6(3):695–708. doi: 10.1534/g3.115.023655 (PMC4777131; doi:10.1534/g3.115.023655)
Supplement: Supporting Information [file supp_6_3_695__index.html]

Major Improvements to the Heliconius melpomene Genome Assembly Used to Confirm 10 Chromosome Fusion Events in 6 Million Years of Butterfly Evolution — Supporting Information 

# Major Improvements to the *Heliconius melpomene* Genome Assembly Used to Confirm 10 Chromosome Fusion Events in 6 Million Years of Butterfly Evolution

## Supporting Information for Davey *et al.*, 2016

**Files in this Data Supplement:**

- Supporting Information - This supplement contains Supporting Methods, legends for supporting figures and tables, and Supporting Figures S1-S8. (.pdf, 1,262 KB)
- Figure S1 - Linkage map construction from raw low coverage genomic SNPs. (.pdf, 68 KB)
- Figure S2 - Stacked bar charts of mean read depths for accepted and rejected SNPs. (.pdf, 144 KB)
- Figure S3 - Ranges of mapped and unmapped region lengths across all Hmel1.1 scaffolds. (.pdf, 133 KB)
- Figure S4 - Length of genome assembly placed on chromosomes (Total) and anchored ordered and oriented, green in Figure 2) on chromosomes (Anchored), for Hmel1.1 and Hmel2. (.pdf, 73 KB)
- Figure S5 - Genome assembly qualities as per Figure 1 for published Lepidopteran genome assemblies. (.pdf, 323 KB)
- Figure S6 - Hmel2 scores against expected scores for Complete and Missing BUSCOs. (.pdf, 291 KB)
- Figure S7 - Hmel2 BUSCO lengths as a percentage of expected BUSCO length for all arthropod BUSCOs. (.pdf, 125 KB)
- Figure S8 - Histogram of differences in size between Hmel1.1 gaps and Hmel2 filled regions. (.pdf, 181 KB)
- File S1 - Supporting methods. (.pdf, 100 KB)
- File S2 - Supporting tables. (.xslx, 504 KB)
- File S3 - Supporting files. (.gz, 96 KB)
