# Supplementary material for: Major Improvements to the Heliconius melpomene Genome Assembly Used to Confirm 10 Chromosome Fusion Events in 6 Million Years of Butterfly Evolution
Source: G3 (Bethesda). 2016 Jan 15;6(3):695–708. doi: 10.1534/g3.115.023655 (PMC4777131; doi:10.1534/g3.115.023655)
Supplement: Supporting Information [file supp_g3.115.023655_FigureS1.pdf]

A

Position	Parent Genotypes FOGM	F1Mo	F1Fa	Offspring Genotypes 1	2	3	4	5	6
79	0/1	1/1	0/0	0/0	0/0	0/1	0/0	0/0	0/0
236	0/0	0/1	0/1	0/1	0/1	1/1	0/1	0/0	0/1
274	0/1	0/0	0/1	0/0	0/0	0/1	0/0	0/0	0/0
409	0/1	0/0	0/1	0/0	0/0	0/1	0/0	0/0	0/0
456	0/0	0/1	0/1	0/1	./.	1/1	0/1	0/0	0/0
540	0/0	0/1	0/1	0/1	0/1	1/1	0/0	0/0	0/1
572	0/1	0/1	0/0	0/1	0/1	0/1	0/1	0/0	0/0
626	0/1	0/0	0/1	./.	0/0	0/1	0/0	0/0	0/0
631	0/1	0/0	0/1	0/0	0/0	0/1	0/0	0/0	0/0
656	1/2	0/2	0/1	0/0	0/1	1/2	0/2	0/1	0/1
660	0/1	0/0	0/1	0/0	0/0	0/1	0/0	0/0	0/1
662	0/1	0/0	0/1	0/0	0/0	0/1	0/0	0/0	0/1
669	0/0	0/1	0/1	0/1	0/1	1/1	0/1	0/0	0/1
674	0/0	0/1	0/1	0/1	0/1	1/1	0/1	0/0	0/1
705	0/1	0/1	0/0	0/1	0/1	0/1	0/1	0/0	0/0
798	0/1	0/1	0/0	0/1	0/1	1/1	0/1	0/0	0/0
819	0/0	0/1	0/1	0/1	0/1	1/1	0/1	0/0	0/1
821	0/1	0/0	0/1	0/0	0/0	0/0	0/0	0/0	0/1
840	0/1	0/0	0/1	0/0	0/0	0/0	0/0	0/0	0/1
842	0/1	0/1	0/0	0/1	0/1	0/1	./.	0/0	0/0
855	0/0	0/1	0/1	0/1	0/1	1/1	0/1	0/0	0/1
937	0/1	0/0	0/1	0/0	0/0	./.	0/0	0/0	0/0
938	0/0	0/1	0/1	0/0	0/1	0/0	0/1	1/1	0/1
986	0/1	0/0	0/1	0/0	0/0	0/1	0/0	0/0	0/1
987	0/1	0/0	0/1	0/0	0/0	0/1	0/0	./.	0/1
1029	0/1	0/0	0/1	0/0	0/0	0/1	0/0	0/0	0/1
1059	0/0	0/1	0/1	0/1	0/1	./.	0/1	0/0	0/0
1121	0/1	0/0	0/1	./.	0/0	0/2	./.	0/0	0/1
1246	0/0	0/1	0/1	0/1	0/1	1/1	0/1	0/0	0/1
1248	0/1	0/1	0/0	0/1	0/1	0/1	0/1	0/0	0/0

B

Parents GMF	Offspring 123456	Type	Reason for rejection
HAB	AABAAA	Reject	Invalid parent call
AHH	HHBHAH	Intercross	
HAH	AAHAAA	Reject	Fails MQ threshold
HAH	AAHAAA	Paternal	
AHH	H.BHAA	Intercross	
AHH	HHBAAH	Intercross	
HHA	HHHHAA	Maternal	
HAH	.AHAAA	Paternal	
HAH	AAHAAA	Paternal	
---	------	Reject	Invalid parent call
HAH	AAHAAH	Paternal	
HAH	AAHAAH	Paternal	
AHH	HHBHAH	Intercross	
AHH	HHBHAH	Intercross	
HHA	HHHHAA	Maternal	
HHA	HHBHAA	Maternal	
AHH	HHBHAH	Intercross	
HAH	AAAAAH	Paternal	
HAH	AAAAAH	Paternal	
HHA	HHH.AA	Maternal	
AHH	HHBHAH	Intercross	
HAH	AA.AAA	Reject	Fails segregation test
AHH	AHBHAH	Intercross	
HAH	AAHAAH	Paternal	
HAH	AAHA.H	Paternal	
HAH	AAHAAH	Paternal	
AHH	HH.HAA	Intercross	
HAH	.AX.AH	Reject	Invalid offspring call 0/2
AHH	HHBHAH	Intercross	
HHA	HHHHAA	Maternal	

C

```
Maternal   Paternal   Intercross
123456     123456     123456
                      ______
                      HHBHAH
           ______     ||||||
           AAHAAA     ||||||
           ||||||     H.BHAA
______     ||||||     HHBAAH
HHHHAA     ||||||     ||||||
||||||     .AHAAA     ||||||
||||||     AAHAAA     ||||||
||||||     |||||_     ||||||
||||||     AAHAAH     ||||||
||||||     AAHAAH     ||||||
||||||      |||||     HHBHAH
||||||      |||||     HHBHAH
HHHHAA      |||||      |||||
HHBHAA      |||||     _|||||
||||||     _|||||     BHBHAH
||||||     HAAAAH     ||||||
||||||     HAAAAH     ||||||
HHH.AA     ||||||     ||||||
||||||     ||||||     BHBHAH
||||||     ||||||     ||||||
||||||     ||||||     AHBHAH
||||||     HAHAAH     ||||||
||||||     HAHA.H     ||||||
||||||     HAHAAH     ||||||
||||||                BH.HAA
||||||                ||||||
||||||                BHBHAH
HHHHAA
```

D

```
Maternal   Paternal   Intercross  Position
123456     123456     123456
                      ______       79
                      HHBHAH       236
           ______     ||||||       274
           AAHAAA     ||||||       409
           ||||||     HHBHAH       456
______     ||||||     HHBHAH       540
HHHHAA     ||||||     ||||||       572
||||||     AAHAAA     ||||||       626
||||||     AAHAAA     ||||||       631
||||||     |||||_     ||||||       656
||||||     AAHAAH     ||||||       660
||||||     AAHAAH     ||||||       662
||||||      |||||     HHBHAH       669
||||||      |||||     HHBHAH       674
HHHHAA      |||||      |||||       705
HHHHAA      |||||     _|||||       798
||||||     _|||||     BHBHAH       819
||||||     HAHAAH     ||||||       821
||||||     HAHAAH     ||||||       840
HHHHAA     ||||||     ||||||       842
||||||     ||||||     BHBHAH       855
||||||     ||||||     ||||||       937
||||||     ||||||     BHBHAH       938
||||||     HAHAAH     ||||||       986
||||||     HAHAAH     ||||||       987
||||||     HAHAAH     ||||||       1029
||||||                BHBHAH       1059
||||||                ||||||       1121
||||||                BHBHAH       1246
HHHHAA                             1248
```

E

Start	End	Maternal	Paternal	Intercross
1	235			
236	408			HHBHAH
409	571		AABAAA	HHBHAH
**572**	**631**	**BBBBAA**	**AABAAA**	**HHBHAH**
632	659	BBBBAA		HHBHAH
**660**	**662**	**BBBBAA**	**AABAAB**	**HHBHAH**
663	674	BBBBAA		HHBHAH
675	818	BBBBAA		
819	820	BBBBAA		BHBHAH
**821**	**1029**	**BBBBAA**	**BABAAB**	**BHBHAH**
1030	1246	BBBBAA		BHBHAH
1247	1248	BBBBAA		
1249	1789			

F

Start	End	Maternal	Paternal	Intercross
1	235			
236	408	**BBBBAA**	**AABAAB**	HHBHAH
409	571	**BBBBAA**	AABAAB	HHBHAH
572	631	**BBBBAA**	**AABAAB**	**HHBHAH**
632	659	**BBBBAA**	**AABAAB**	**HHBHAH**
660	662	**BBBBAA**	**AABAAB**	**HHBHAH**
663	674	BBBBAA	**AABAAB**	HHBHAH
675	818	BBBBAA		
819	820	BBBBAA	**BABAAB**	BHBHAH
821	1029	BBBBAA	BABAAB	BHBHAH
1030	1246	BBBBAA	**BABAAB**	BHBHAH
1247	1248	BBBBAA		
1249	1789			

G

Start	End	Maternal	Paternal	Intercross	Chromosome	cM
1	235				-	-
236	674	BBBBAA	AABAAB	HHBHAH	18	1.45
675	818	BBBBAA			18	-
819	1246	BBBBAA	BABAAB	BHBHAH	18	2.889
1247	1248	BBBBAA			18	-
1249	1789				-	-

Figure S1

Figure S1 Linkage map construction from raw low coverage genomic SNPs. The figure shows the process of calling markers for one scaffold region from raw SNPs (A) to final chromosome and marker assignment (G). See Supplementary Methods for full discussion. (A) Raw SNPs for 30 positions with GATK VCF calls for parents (F0GM, F0 Grandmother; F1Mo, F1 Mother; F1Fa, F1Father) and offspring (1-6; in the actual study, 69 offspring were present). (B) Assignment of SNPs to marker types. G=F0 Grandmother, M=F1 Mother, F=F1 Father. A=homozygous for allele A, B=homozygous for allele B, H=heterozygous for A and B, '.'=missing, '-',X=invalid. (C) Separation of genotypes into marker types and grouping of SNPs into marker sub-regions. Marker sub-regions are bound by horizontal black lines and connected by vertical grey lines. Erroneous genotypes (at this point unknown to the algorithm) in red. (D) Consensus genotypes for all sub-regions. Bold, imputed or corrected genotypes. (E) Collapsed marker regions. Regions with maternal, paternal and intercross markers shown in bold; consistent genotypes in green, inconsistent genotypes in red. (F) Corrected and imputed marker regions. (G) Final collapsed maternal, paternal and intercross marker regions used to build linkage map, with resulting chromosome and cM marker assignments.
