# Supplementary material for: Major Improvements to the Heliconius melpomene Genome Assembly Used to Confirm 10 Chromosome Fusion Events in 6 Million Years of Butterfly Evolution
Source: G3 (Bethesda). 2016 Jan 15;6(3):695–708. doi: 10.1534/g3.115.023655 (PMC4777131; doi:10.1534/g3.115.023655)
Supplement: Supporting Information [file supp_g3.115.023655_FigureS2.pdf]

Figure S2

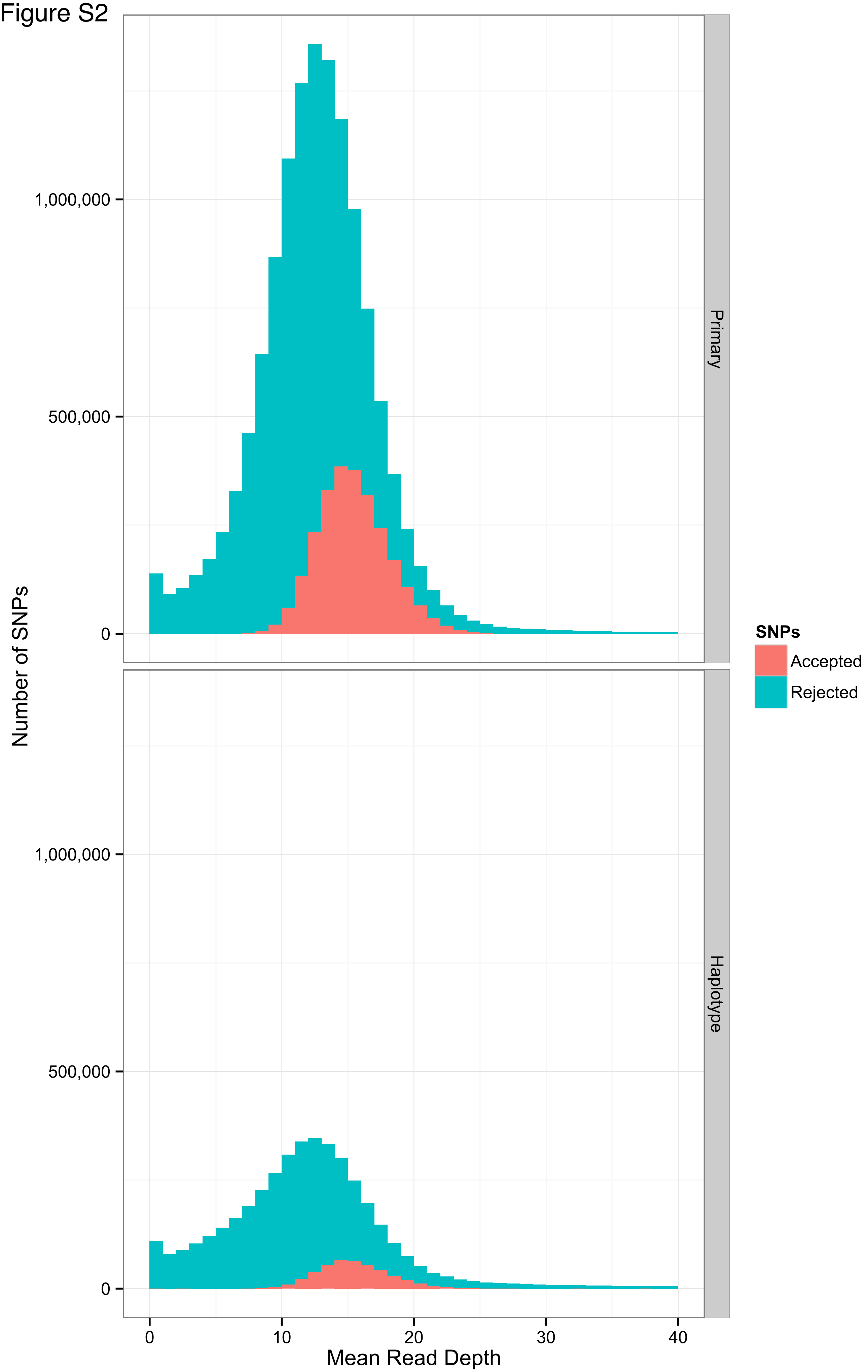

Primary
Haplotype
1,000,000
500,000
0
1,000,000
500,000
0
0
10
20
30
40
Mean Read Depth
Number of SNPs
SNPs
Accepted
Rejected

Figure S2 Stacked bar charts of mean read depths for accepted and rejected SNPs. Read depths calculated per marker, taking a mean for each marker across all individuals. Distributions truncated at maximum 40 reads; maximum read depth was 427.
