# Supplementary material for: Major Improvements to the Heliconius melpomene Genome Assembly Used to Confirm 10 Chromosome Fusion Events in 6 Million Years of Butterfly Evolution
Source: G3 (Bethesda). 2016 Jan 15;6(3):695–708. doi: 10.1534/g3.115.023655 (PMC4777131; doi:10.1534/g3.115.023655)
Supplement: Supporting Information [file supp_g3.115.023655_FigureS3.pdf]

Figure S3

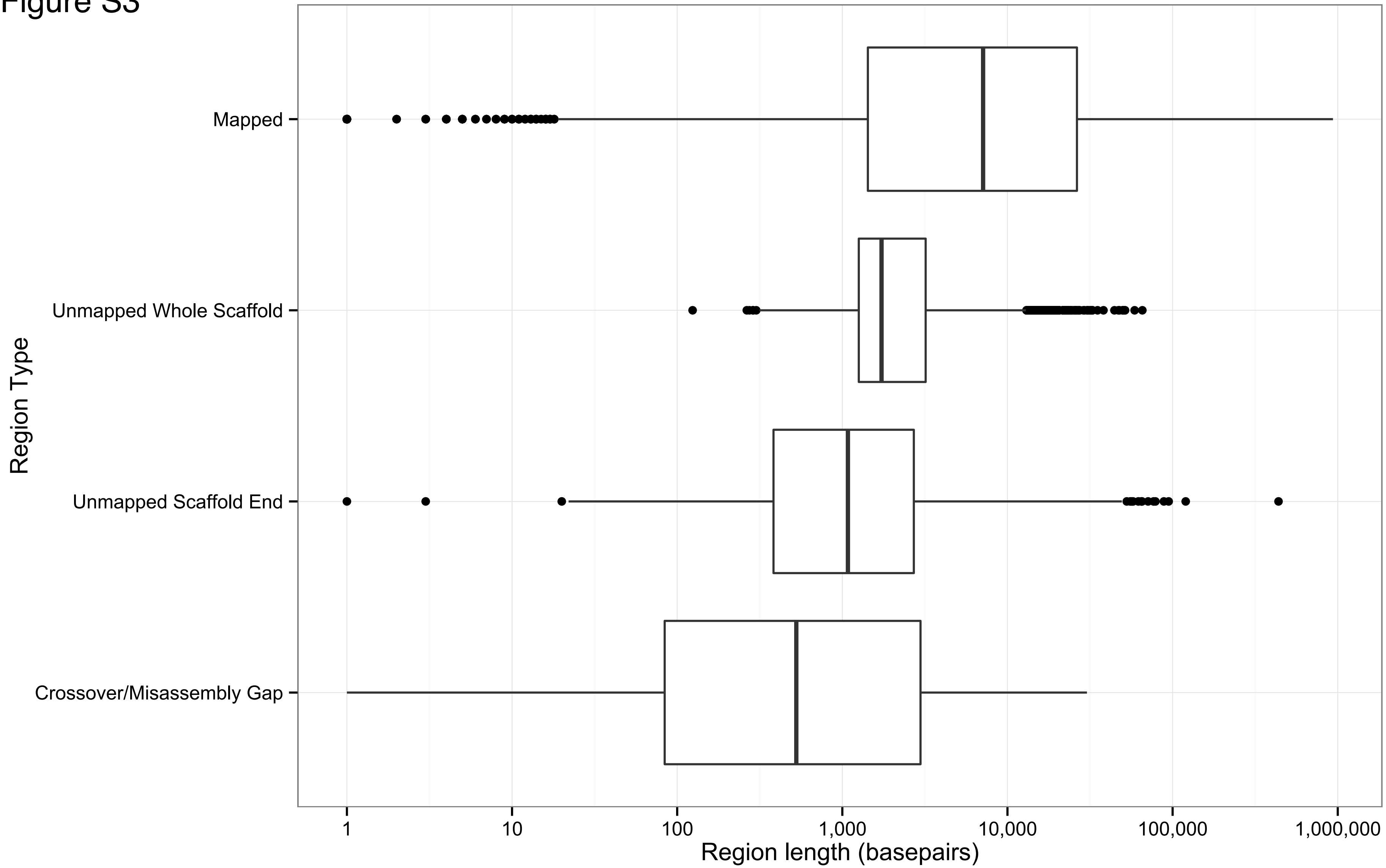

Mapped
Unmapped Whole Scaffold
Unmapped Scaffold End
Crossover/Misassembly Gap
Region Type
1
10
100
1,000
10,000
100,000
1,000,000
Region length (basepairs)

Figure S3 Ranges of mapped and unmapped region lengths across all Hmel1.1 scaffolds. Crossover/ Misassembly Gaps occur within scaffolds between markers, either consecutive on one chromosome (Crossover) or distant on one chromosome or on different chromosomes (Misassembly).
