# Supplementary material for: Major Improvements to the Heliconius melpomene Genome Assembly Used to Confirm 10 Chromosome Fusion Events in 6 Million Years of Butterfly Evolution
Source: G3 (Bethesda). 2016 Jan 15;6(3):695–708. doi: 10.1534/g3.115.023655 (PMC4777131; doi:10.1534/g3.115.023655)
Supplement: Supporting Information [file supp_g3.115.023655_FigureS4.pdf]

Figure S4

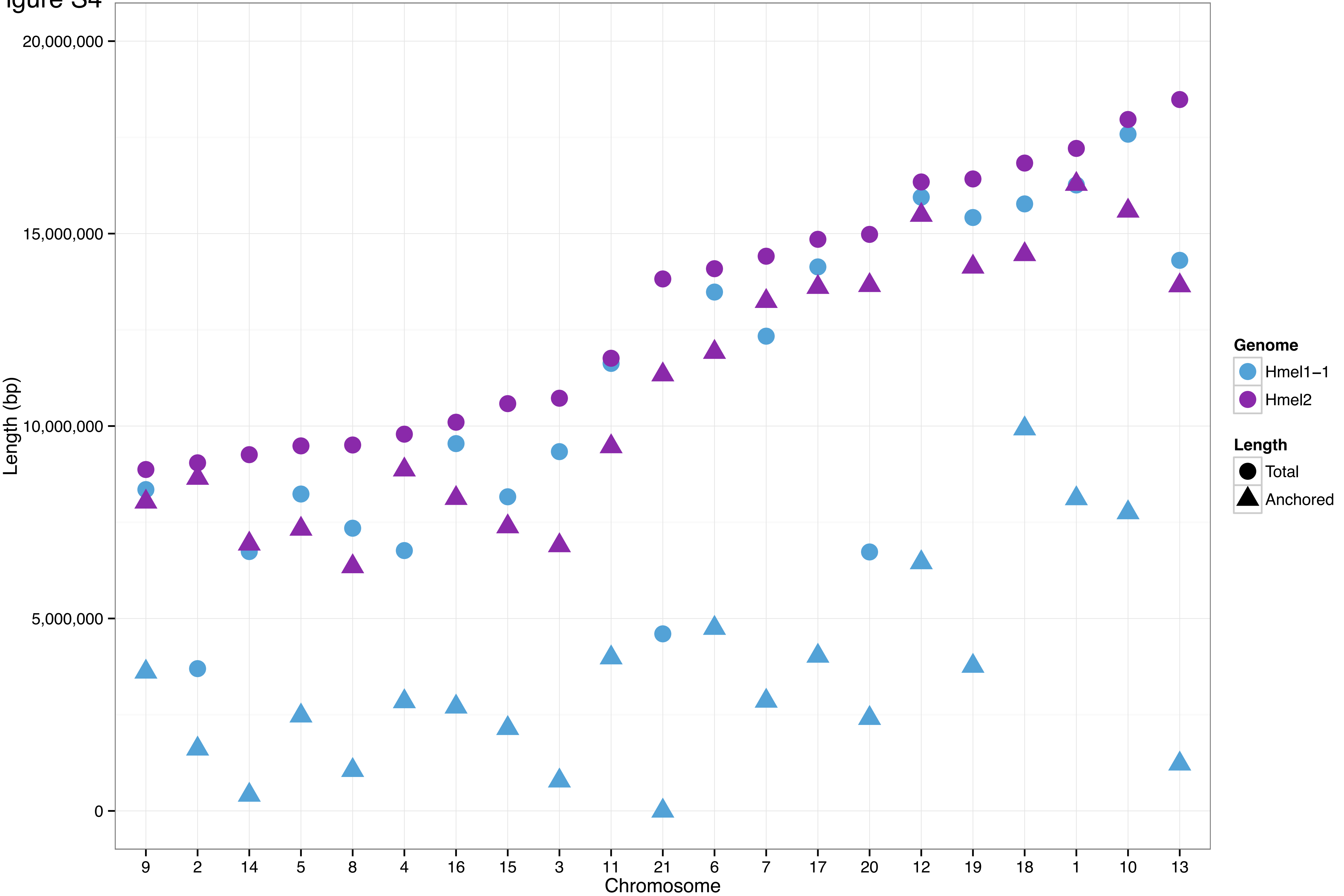
20,000,000
15,000,000
10,000,000
5,000,000
0
Length (bp)
9
2
14
5
8
4
16
15
3
11
21
6
7
17
20
12
19
18
1
10
13
Chromosome
Genome
Hmel1−1
Hmel2
Length
Total
Anchored

Figure S4 Length of genome assembly placed on chromosomes (Total) and anchored (ordered and oriented, green in Figure 2) on chromosomes (Anchored), for Hmel1.1 and Hmel2. Chromosomes ordered by total length in Hmel2.
