# Supplementary material for: Major Improvements to the Heliconius melpomene Genome Assembly Used to Confirm 10 Chromosome Fusion Events in 6 Million Years of Butterfly Evolution
Source: G3 (Bethesda). 2016 Jan 15;6(3):695–708. doi: 10.1534/g3.115.023655 (PMC4777131; doi:10.1534/g3.115.023655)
Supplement: Supporting Information [file supp_g3.115.023655_FigureS5.pdf]

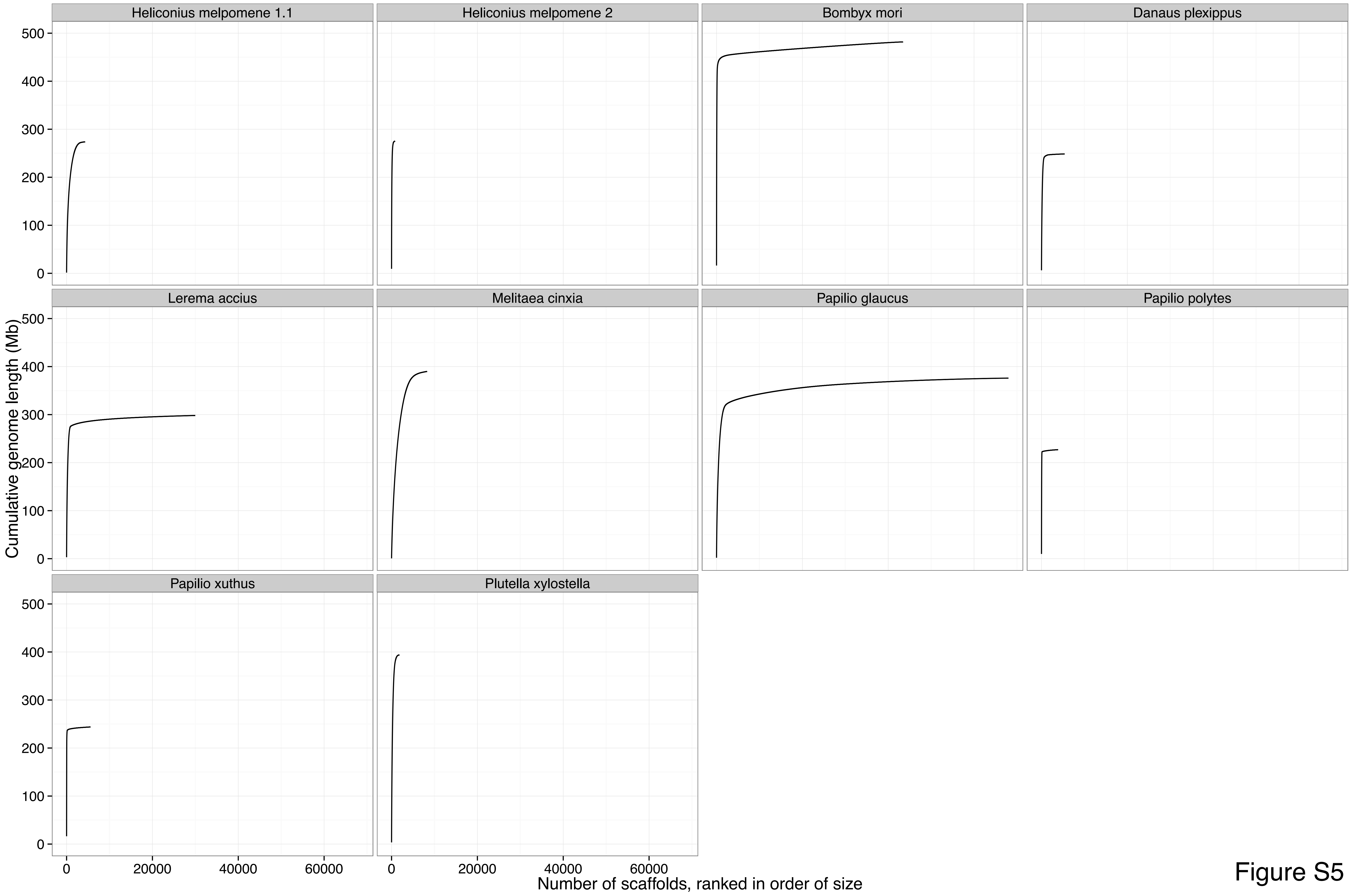
Heliconius melpomene 1.1
Heliconius melpomene 2
Bombyx mori
Danaus plexippus
Lerema accius
Melitaea cinxia
Papilio glaucus
Papilio polytes
Papilio xuthus
Plutella xylostella
0
100
200
300
400
500
0
20000
40000
60000
Cumulative genome length (Mb)
Number of scaffolds, ranked in order of size

Figure S5

Figure S5 Genome assembly qualities as per Figure 1 for published Lepidopteran genome assemblies.
