# Supplementary material for: Major Improvements to the Heliconius melpomene Genome Assembly Used to Confirm 10 Chromosome Fusion Events in 6 Million Years of Butterfly Evolution
Source: G3 (Bethesda). 2016 Jan 15;6(3):695–708. doi: 10.1534/g3.115.023655 (PMC4777131; doi:10.1534/g3.115.023655)
Supplement: Supporting Information [file supp_g3.115.023655_FigureS6.pdf]

Figure S6

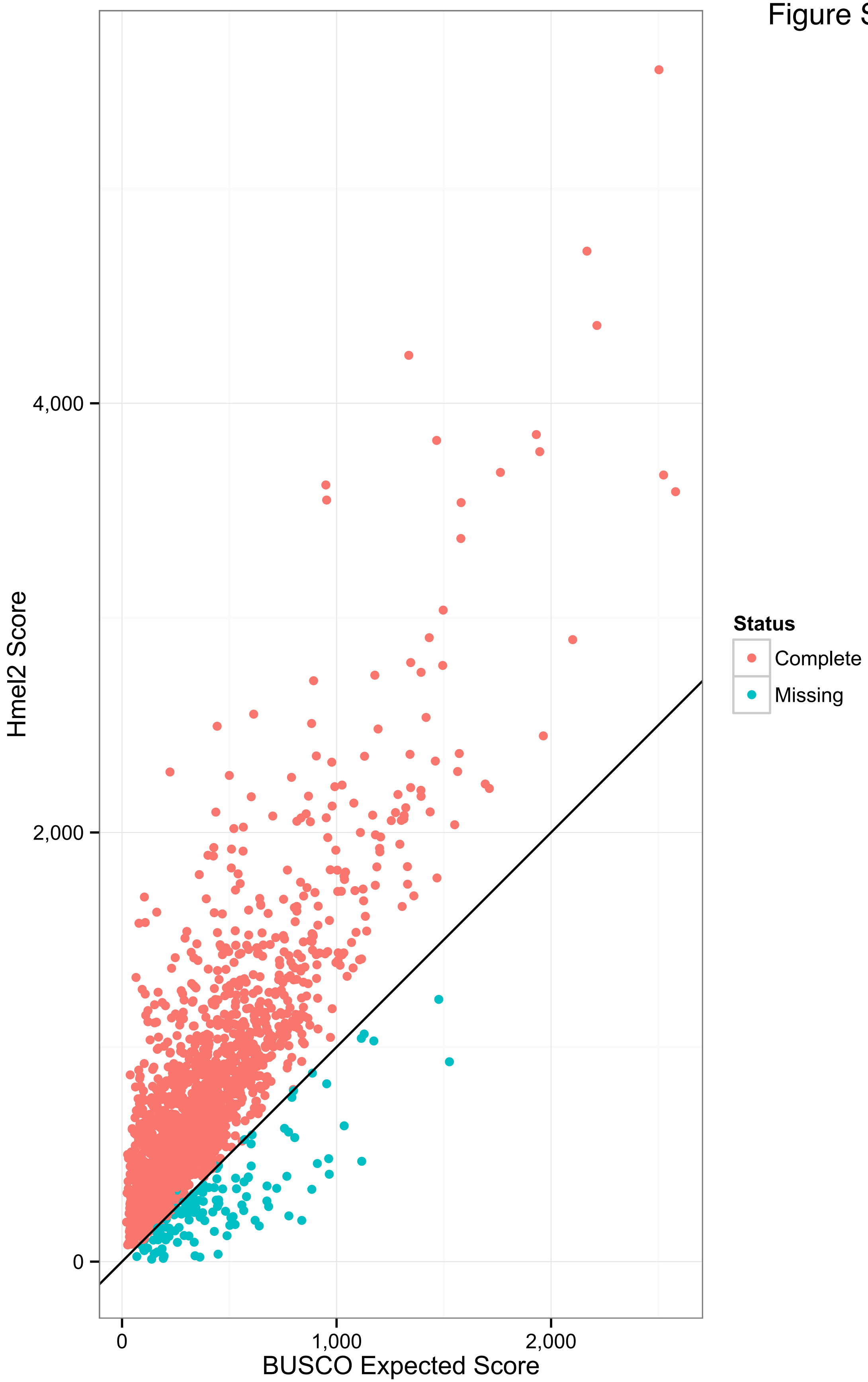

Hmel2 Score
4,000
2,000
0
0
1,000
2,000
BUSCO Expected Score
Status
Complete
Missing

Figure S6 Hmel2 scores against expected scores for Complete and Missing BUSCOs. Each of 2675 arthropod BUSCOs has an expected score and expected length calculated across 38 arthropod genomes. BUSCOs with Hmel2 scores below the expected score are labelled Missing (black line, score threshold). Duplicated and Fragmented scores not shown for clarity.
