# Supplementary material for: Major Improvements to the Heliconius melpomene Genome Assembly Used to Confirm 10 Chromosome Fusion Events in 6 Million Years of Butterfly Evolution
Source: G3 (Bethesda). 2016 Jan 15;6(3):695–708. doi: 10.1534/g3.115.023655 (PMC4777131; doi:10.1534/g3.115.023655)
Supplement: Supporting Information [file supp_g3.115.023655_FigureS7.pdf]

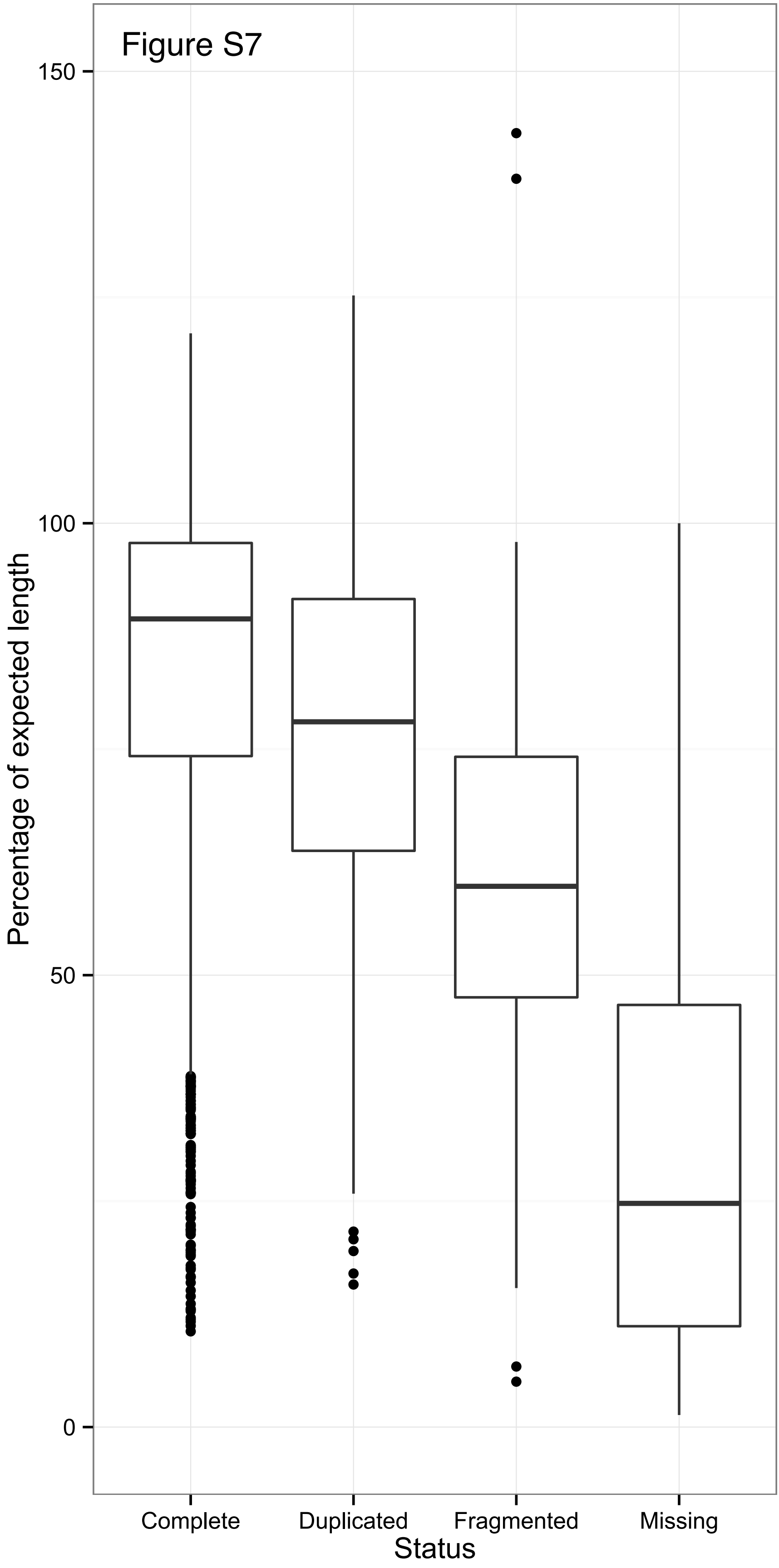
Figure S7
150
100
50
0
Percentage of expected length
Complete
Duplicated
Fragmented
Missing
Status

Figure S7 Hmel2 BUSCO lengths as a percentage of expected BUSCO length for all arthropod BUSCOs.
