# Supplementary material for: Major Improvements to the Heliconius melpomene Genome Assembly Used to Confirm 10 Chromosome Fusion Events in 6 Million Years of Butterfly Evolution
Source: G3 (Bethesda). 2016 Jan 15;6(3):695–708. doi: 10.1534/g3.115.023655 (PMC4777131; doi:10.1534/g3.115.023655)
Supplement: Supporting Information [file supp_g3.115.023655_FigureS8.pdf]

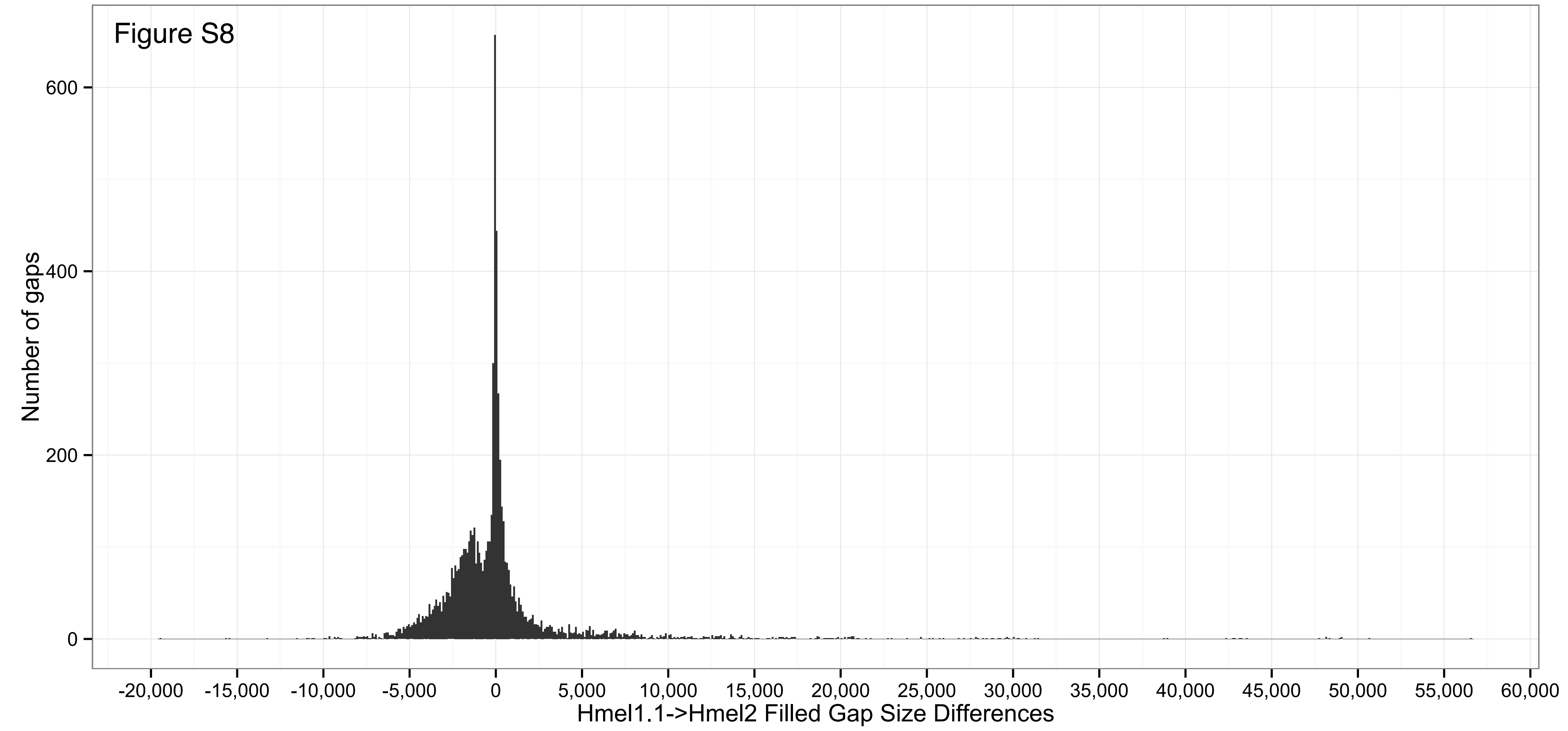

Figure S8
Number of gaps
600
400
200
0
-20,000
-15,000
-10,000
-5,000
0
5,000
10,000
15,000
20,000
25,000
30,000
35,000
40,000
45,000
50,000
55,000
60,000
Hmel1.1->Hmel2 Filled Gap Size Differences

Figure S8 Histogram of differences in size between Hmel1.1 gaps and Hmel2 filled regions. Positive values indicate increase in size in Hmel2.
