# Supplementary material for: Major Improvements to the Heliconius melpomene Genome Assembly Used to Confirm 10 Chromosome Fusion Events in 6 Million Years of Butterfly Evolution
Source: G3 (Bethesda). 2016 Jan 15;6(3):695–708. doi: 10.1534/g3.115.023655 (PMC4777131; doi:10.1534/g3.115.023655)
Supplement: Supporting Information [file supp_g3.115.023655_FileS1.pdf]

File S1. Supporting Methods

This supplement explains our assembly design and describes the construction of linkage maps from raw low coverage genomic SNPs. The process is summarised in Figure S1. This figure shows an example scaffold region with 30 SNPs, with calls for 6 offspring, simplified for explanation. The real data set has 69 offspring calls and has many more rejected SNPs than accepted SNPs (see Table S2 for counts of SNPs). Full data and code can be found in the Dryad and GitHub repositories associated with the paper and is referred to below where appropriate (see also Supplementary Files for code).

Linkage mapping and assembly strategy

Following the publication of Hmel1.1 (Heliconius Genome Consortium, 2012), we wished to further improve the contiguity of, and reduce misassemblies in, the *H. melpomene* genome. Given the success of linkage mapping using RAD sequencing for Hmel1.1, we decided to extend this approach to whole genome sequencing for Hmel2, aiming to achieve very detailed coverage across the vast majority of the genome and allowing us to include many more scaffolds on the linkage map, while correcting many hundreds of misassemblies to within a few hundred or thousand bases. A whole genome sequencing strategy requires a trade off between coverage per individual and number of individuals given available funds. We wished to increase the number of individuals contributing to the map from the 43 individuals RAD sequenced for Hmel1.1, but aimed to achieve decent genotyping for as many individuals as possible. We decided to aim to sequence ~10x coverage of ~70 individuals based on available funds and available DNA from remaining mapping cross larvae. 10x coverage is not sufficient to reliably genotype one SNP in one individual. However, we only needed to call maternal and paternal markers accurately, not each SNP, and we expected to find tens to hundreds of SNPs for each marker, given a

previously published *H. melpomene* recombination rate estimate of 180 kb/cM (Jiggins *et al.* 2005) and ~1% heterozygosity. The process of collapsing tens of millions of raw SNPs to unique maternal and paternal markers required bespoke analysis, as existing linkage mapping software can only handle tens of thousands of markers at most, and is often highly sensitive to genotyping errors, which can be difficult to distinguish from genuine recombinations. The pipeline we used to collapse SNPs to markers is described below.

Sequencing 70 individuals does not give us enough recombinations to completely resolve scaffold order and orientation along the genome, as many scaffolds were smaller than the recombination rate expected for this number of *H. melpomene* individuals (~250 kb/cM). The final Hmel2 map still has many loci with unordered or unoriented scaffolds. However, we attempted to alleviate this problem by sequencing *H. melpomene* with Pacific Biosciences sequencing and increasing scaffold lengths by assembling the PacBio reads and incorporating them into the genome. This, combined with incorporating our previously discarded haplotype scaffolds, extended the contiguity of the genome substantially even prior to placing scaffolds on the linkage map and allowed many more regions of the genome to be ordered and oriented than would have been possible with the linkage map data alone.

Filters and parameters were chosen empirically; for linkage mapping, this meant balancing between maximising the number of scaffolds incorporated on the map and producing accurate linkage maps; for genome scaffolding; this meant maximising contiguity and minimising gene loss, gene breakage and the number of scaffolds. Comparing to a linkage map is a powerful way of detecting misassemblies introduced by overzealous merging of scaffolds, which we could detect in HaploMerger and FALCON outputs by mapping linkage map markers to the resulting assemblies.

SNP validation and conversion to segregation patterns

Raw SNPs produced by GATK are given in the format (0/0, 0/1, 1/1), where 0 is the reference allele, 1 is the alternate allele, 0/0 is a reference homozygote, 1/1 is an alternate homozygote and 0/1 is a heterozygote (Figure S1A; see Hmel_cross.Hmel1-1_primaryScaffolds.vcf.gz and Hmel_cross.Hmel_haplotype_scaffolds.vcf.gz in the Dryad repository). We used these raw SNPs to infer linkage map markers for our cross. Typically it is possible to produce a maternal and paternal linkage map from one F2 cross. Because recombination is absent in Lepidopteran females (Turner and Sheppard 1975), the true maternal map consists of only 21 markers, one for each of the 21 *H. melpomene* chromosome, known as chromosome prints. We expect to find several tens of true paternal markers for each chromosome. A SNP can be labelled as contributing to the maternal or paternal map based on the parental calls and how they segregate in the offspring. Maternal markers can be identified where the mother is heterozygous and the father is homozygous; paternal markers, where the mother is homozygous and the father is heterozygous. If the offspring show appropriate Mendelian segregation for homozygous and heterozygous calls, this segregation will be due to the heterozygous parent, and the SNP can be assigned to the maternal or paternal map.

We redefined GATK genotypes to a single character code, where A was homozygous for allele 0 (eg 0/0), B was homozygous for allele 1 (eg 1/1) and H was heterozygous (0/1) (Figure S1B). We then defined a series of marker types which are valid for an F2 intercross (Table S2). These marker types do not cover all possible segregations in an F2 cross, only those that are informative for linkage mapping. For example, 6.9 million SNPs are rejected because their parent call is invalid, but 3.5 million of these are rejected

because both parents are homozygous for the same allele. Such SNPs will not be informative for linkage mapping and are probably genuine homozygous sites incorrectly called as SNPs due to an incorrect genotype in one or a few offspring. A valid type would be, for example, if the mother is heterozygous (H) and the father is (A), where we expect all offspring to segregate equally for homozygote A and heterozygote H, regardless of sex (top row of Table S2). As we had not only the F1 mother and F1 father but also the F0 grandmother available, we could require consistent genotypes from all three parental samples as well as all offspring.

The assignment of SNPs to maternal and paternal groups is complicated in an F2 cross by two factors. Firstly, segregation on the sex chromosomes differs to that on the autosomes, requiring special marker types for Z-linked markers and for pseudo-autosomal regions (shown in Table S2). Secondly, a full-sib cross produces many regions of the genome where the two F1 parents inherit the same chromosomes from the F0 grandparents, and so both parents can have the same heterozygous SNPs (these SNPs have been labelled Intercross). These SNPs segregate in a 1:2:1 ratio (shown as A, 2H, B in Table S2) and can not be assigned to either the maternal or paternal map initially, as it is unclear which parent passed on which alleles for each offspring. Fortunately, it is possible to separate these intercross markers into maternal and paternal markers once a set of candidate maternal and paternal markers have been identified (see below).

All SNPs were assigned to a marker type from Table S2 according to the calls for the two F1 parents and F0 grandmother or rejected if the parental genotypes did not match any of these valid marker types (Figure S1B). SNPs assigned to a marker type were rejected if any offspring had an invalid call for the assigned marker type (for example, a homozygous B call where the parents have A and H calls) or if the offspring calls failed a root-mean-

square test for goodness of fit to expected segregation for the marker type (Perkins *et al.* 2011); however, missing genotypes were allowed.

Quality thresholds were applied to the remaining SNPs based on metrics reported by GATK. SNPs were rejected if parental sequencing depth was greater than 85 reads for any parental call; if the SNP had FS (Fisher Strand bias) value greater than 5; if the SNP had MQ (Mapping Quality) value less than 90; or if parental genotype quality fell below 99 for heterozygous calls or 60 for homozygous calls. The last condition was imposed because GATK produces systematically lower qualities for homozygous calls than heterozygous calls in low coverage data, on the assumption that the alternate allele may not have been sequenced. These quality thresholds were defined empirically by balancing the number of scaffolds placed on the map and the coverage of each placed scaffold with accuracy of the resulting linkage map. The effectiveness of the filters can be seen in Figure S2, showing the number of accepted and rejected SNPs and their mean coverages.

The number of SNPs accepted for each marker type and rejected by each filter is shown in Table S2. Accepted SNPs were then grouped by marker type and calls for all offspring were concatenated into one string to form a segregation pattern (eg offspring with calls A, H, H, A, H have segregation pattern AHHAH; Figure S1C). Calls were phased across scaffolds for each marker type to ensure segregation patterns of each type could be compared (for example, two consecutive SNPs with segregation patterns AHHAH and HAAHA were phased so both were AHHAH; this is not shown in Figure S1). All of this information is presented for all SNPs in the ‘markers’ table in Hmel_cross.linkage_map.db in the Dryad repository. This database was generated by scaffoldgenome.pl (see Supplementary Files for details).

Identifying marker regions

In principle, it would be possible to take the 2.9 million accepted segregation patterns at this point and build a linkage map with them directly. However, no existing linkage map software can handle this number of markers, and many missing and erroneous genotypes remain; although all the offspring genotypes are valid for the marker type and appear to segregate appropriately, they are not necessarily correct (for example, an accepted pattern with A and H valid offspring genotypes will not have offspring with B calls but could have some offspring with incorrect A or H calls). Therefore, it was necessary to reduce the number of segregation patterns before building linkage maps.

Because SNPs are aligned to the existing reference genome, consecutive SNPs along a scaffold with the same marker type and the same segregation pattern can be collapsed to a single marker, and the region of the scaffold bounded by this group of SNPs can be labelled a marker region for this marker (Figures S1D, S1E). By comparing SNPs within such a region, errors can be corrected, missing genotypes imputed, and recombinations detected.

Scaffolds were split into different candidate marker regions if more than 25% of offspring differed in their genotype between two consecutive SNPs (Figures S1C and S1D show one such candidate marker region). For each offspring, the defined scaffold regions were then split into sub-regions by consecutive identical genotype calls, rejecting sub-regions shorter than 100bp (likely due to mis-mapping or poor quality reads). Figure S1C shows sub-regions for each offspring (1-6) bound by black horizontal lines, with grey vertical lines showing connections along sub-regions. Offspring 2-6 have the same sub-regions, but offspring 1 is split into two sub-regions for the Paternal and Intercross markers due to a

recombination somewhere between positions 674 and 819. Missing and erroneous genotypes are not split into separate sub-regions.

Consensus genotypes could then called for each offspring along each sub-region. These sub-region consensus genotypes were then used to call genotypes for the whole marker region (ie all the SNPs in Figure S1C), allowing at most one recombination in paternal and intercross markers and no recombinations in maternal markers. Figure S1D shows consensus genotypes for the calls in Figure S1C, with corrected and imputed genotypes in bold. This procedure, carried out for each individual separately, sometimes produces incorrect calls, such as the paternal call for offspring 6, highlighted in red in Figure S1D. Consensus marker regions are presented in the ‘consensus’ column of the ‘markers’ table in Hmel_cross.linkage_map.db in the Dryad repository and generated by scaffoldgenome.pl (see Supplementary Files for details).

Identification of maternal chromosome prints and paternal markers

Consensus marker regions can now be collapsed as shown in Figure S1E; these regions are presented in the ‘blocks’ table in Hmel_cross.linkage_map.db in the Dryad repository, generated by scaffoldgenome.pl (see Supplementary Files for details). For maternal and paternal markers, H has now been replaced by B, as, for example, a heterozygous call in a maternal marker means the offspring inherited allele B from the mother. This makes it possible to see where intercross markers are consistent with maternal and paternal markers (Maternal A+ Paternal A=Intercross A, B+B=B, A+B=H). The regions at 660 and 821 (in green) are consistent, but the region at 572 (with inconsistent offspring 6 genotypes highlighted in red) is not consistent. Consistency across the three marker types indicates the marker is likely to be valid, although the same error can occur across marker types in small regions.

The 21 chromosome prints for Hmel2 were identified by finding marker regions with consistent maternal, paternal and intercross markers and then extracting the set of maternal markers across the whole genome. This produced several hundred unique maternal markers, with the real markers covering large regions of the genome and errors covering small regions. To collapse errors and identify the chromosome prints, log odds (LOD) scores were calculated between each pair of maternal markers and, if a pair of markers had a LOD score below 6, the markers were joined together into one print. 19 of 21 chromosome prints could be identified in this way. Given these 19 valid maternal markers, the set of consistent marker regions could then be increased by correcting the erroneous maternal markers. Assuming these consistent markers are valid, other scaffold regions that had only a paternal or intercross marker (for example, at position 236) could be assigned maternal and paternal markers where they matched a marker in the consistent set (Figure S1F). Inconsistent marker regions could also be corrected by checking against this consistent set (eg block 572-631 in Figure S1E-F; although the consistent block is very small in this example here, in most real cases the same block appears on other scaffolds featuring the same marker, supporting its correctness).

Only 19 of 21 maternal chromosome prints could be identified using this method. Two chromosomes segregated identically in both F1 parents and so only produced intercross markers, because both parents shared the same chromosomes and so the same variants, meaning both parents were heterozygous at all real SNPs. These chromosome prints were identified by collapsing all remaining intercross markers without matching maternal markers into sets of markers with 6 or fewer different homozygous calls and calculating a consensus of homozygous calls for each set. This produced two sets each with one consensus marker, representing the two missing chromosomes. Paternal markers for

regions with one of these markers could then be inferred from the intercross and maternal markers together. This produced a set of 21 maternal chromosome prints and a set of consistent paternal markers with assignments to a maternal marker and to regions across all scaffolds (Figure S1F-G; S1G shows the intercross markers for clarity, although they are no longer used from this point, as they can all be converted to paternal markers). The corresponding real data for Figure S1F is found in the 'cleanblocks' table in Hmel_cross.linkage_map.db in the Dryad repository, and for Figure S1G in the 'mapblocks' table in the same database. The 'cleanblocks' table is generated by the clean_blocks.pl and 'mapblocks' by build_linkage_maps.pl (see Supplementary Files for details). At this point, many errors have been corrected, but some still remain and need to be filtered during linkage map construction.

Linkage map construction

Linkage maps were constructed for each chromosome by ordering paternal markers assigned to each of the 21 maternal chromosome prints iteratively using MSTMap (Wu *et al.* 2008). We used MSTMap because it can be run from a script iteratively and rapidly, allowing us to add markers to the maps incrementally and checking for their accuracy as they were added. MSTMap was run with the following options: population_type RIL2, distance_function kosambi, cut_off_p_value 0.000001, no_map_dist 0, no_map_size 0, missing_threshold 1, estimation_before_clustering yes, detect_bad_data yes, objective_function ML.

The script build_linkage_maps.pl (see Supplementary Files) was used to construct the linkage maps. For each chromosome, an initial map was built using paternal markers each covering more than 200,000 base pairs. MSTMap sometimes returned 2 or more linkage groups due to different phasing across scaffolds; for example, one scaffold may have a

marker AABAAB and another marker BBABBA, which represent the same marker. If this occurred, paternal markers were phased to match the first linkage group and the map was built again to produce a single linkage group. Remaining paternal markers were then ordered by the number of base pairs they covered, largest first, and added to the map one by one, rebuilding the map each time. If the new marker was incorporated and introduced a double recombination at that marker in one offspring, that offspring was corrected and the marker was merged into the correct neighbouring marker. If the new marker created a disordered map, or it was added at either end of the map, or it could not be incorporated at all, it was rejected. After all markers had been processed once, further attempts were made to incorporate the rejected markers using the same rules, until an iteration through all remaining markers added no new markers to the map.

We believe the markers contained on the resulting maps are accurate and correctly ordered. However, it may be that accurate markers have been discarded, particularly at the ends of chromosomes, although manual inspection of the rejected markers did not reveal any obviously correct discarded markers. Nevertheless, even if the map is correct, errors remained in the assignment of markers to scaffolds. Consider two correct neighbouring markers AABAAB and BABAAB, with a recombination in offspring 1. These markers may have been accurately assigned to the appropriate scaffold regions for the most part, and so these scaffolds can be assigned to the linkage map (as shown in Figure S1G). However, there may be a small scaffold region with incorrect genotypes for offspring 1 that appears to be, say, AABAAB when it is in fact BABAAB. A correct marker may therefore be assigned to an incorrect location. Cleaning up these incorrect calls for valid markers was done through manual inspection during the merging process, as described in the main Methods section. The final map ('chromosome_map' table) and cleaned marker

regions (‘scaffold_map’ table) can be found in the Hmel_cross.linkage_map.clean.db database in the Dryad repository.

For any one scaffold, regions with maternal and paternal markers can be assigned a chromosome and cM position (236 and 1247 in Figure S1G), regions with maternal markers only can be assigned a chromosome (675 and 1247) and regions with no markers are left unassigned, including the starts and ends of every scaffold.
